# Analysis of ParAB dynamics in mycobacteria shows active movement of ParB and differential inheritance of ParA

**DOI:** 10.1371/journal.pone.0199316

**Published:** 2018-06-19

**Authors:** Iria Uhía, Miles Priestman, Graham Joyce, Nitya Krishnan, Vahid Shahrezaei, Brian D. Robertson

**Affiliations:** 1 MRC Centre for Molecular Bacteriology and Infection, Department of Medicine, Imperial College London, London, United Kingdom; 2 Department of Mathematics, Imperial College London, London, United Kingdom; Bose Institute, INDIA

## Abstract

Correct chromosomal segregation, coordinated with cell division, is crucial for bacterial survival, but despite extensive studies, the mechanisms underlying this remain incompletely understood in mycobacteria. We report a detailed investigation of the dynamic interactions between ParA and ParB partitioning proteins in *Mycobacterium smegmatis* using microfluidics and time-lapse fluorescence microscopy to observe both proteins simultaneously. During growth and division, ParB presents as a focused fluorescent spot that subsequently splits in two. One focus moves towards a higher concentration of ParA at the new pole, while the other moves towards the old pole. We show ParB movement is in part an active process that does not rely on passive movement associated with cell growth. In some cells, another round of ParB segregation starts before cell division is complete, consistent with initiation of a second round of chromosome replication. ParA fluorescence distribution correlates with cell size, and in sister cells, the larger cell inherits a local peak of concentrated ParA, while the smaller sister inherits more homogeneously distributed protein. Cells which inherit more ParA grow faster than their sister cell, raising the question of whether inheritance of a local concentration of ParA provides a growth advantage. Alterations in levels of ParA and ParB were also found to disturb cell growth.

## Introduction

The ParABS system was originally described in the segregation of low-copy number plasmids, but homologous proteins have been identified in many bacteria, including the genus *Mycobacterium* [1–8], where they participate in chromosome partitioning [9]. Understanding how chromosomes are segregated in mycobacteria is of critical importance, with the increase in *Mycobacterium tuberculosis* drug-resistant strains requiring the urgent development of novel therapeutics [10].

ParB is a site-specific DNA-binding protein that binds with high affinity to *parS* sequence motifs, usually clustered near the origin of replication, *oriC* [1,2,11,12]. ParA is a Walker-type ATPase that binds non-specifically to DNA but also interacts with ParB. DNA segregation mediated by the ParABS system has been extensively studied in *Caulobacter crescentus* [13,14]. Briefly, ParB binds specifically to the *parS* sites, forming centromere-like complexes [15]. ParA dimerises in the presence of ATP and binds non-specifically to DNA. ParB-*parS* complexes interact with ParA dimers and stimulate ParA ATPase activity causing ParA to dissociate from the nucleoid. This dissociation is proposed to trigger ParB-*parS* movement towards the area of highest ParA concentration, the new pole in *C*. *crescentus*, thus segregating the replicated chromosome [14]. This new pole-specific directionality is thought to be conferred by PopZ and TipN proteins [13,14]. The precise mechanism coupling ParA monomerisation with ParB-*parS* movement is unclear, nor is it known how the ParA gradient is formed. Different models have been proposed to answer these questions [14,16–18], but irrespective of the model, the outcome is the same: a fraction of ParB (presumably bound to one of the newly replicated chromosomes) moves towards the new cell pole, whereas the remaining ParB is bound to the other chromosome and stays close to the old pole.

In mycobacteria, ParB dynamics [7,8] are similar to those observed in *C*. *crescentus* and in other actinobacteria, such as *Corynebacterium glutamicum* [19], with the difference that in mycobacteria a single ParB focus is usually localised near midcell, instead of near to the old pole, as observed in *Caulobacter* and *Corynebacterium*. The ParB focus duplicates, and the two foci segregate towards opposing quarter-cell positions, each of them being subsequently inherited by a daughter cell [7,8]. ParB has been found to participate in replisome positioning [7,8] and septum placement [8].

ParA and ParB proteins are essential in *M*. *tuberculosis* [20,21], but not in *M*. *smegmatis*. However, their overproduction or deletion causes growth retardation, aberrations in cell length and chromosome segregation defects [1,3,6]. During preparation of this manuscript a study was published on ParAB dynamics, reporting similar results to those described here [22]. In this report we used time-lapse microscopy with ParA and ParB fluorescent reporters to observe the dynamics of chromosome replication and segregation in *M*. *smegmatis*. As well as deciphering ParA-ParB choreography, our major findings include novel aspects of the behaviour of these proteins such as the observation of active movement of ParB at certain distances from ParA, and unequal inheritance of ParA in sister cells, with the larger sister inheriting an area of concentrated ParA, whereas the smaller sister inherits more homogeneously distributed protein. Our data highlight the importance of ParABS in chromosome segregation in mycobacteria, and show that movement of the chromosome partitioning machinery is a highly organised process.

## Results

### Characterisation of *M*. *smegmatis* Δ*parAB* [pMEND-AB] for the study of ParA-ParB dynamics

**Fig 1:**
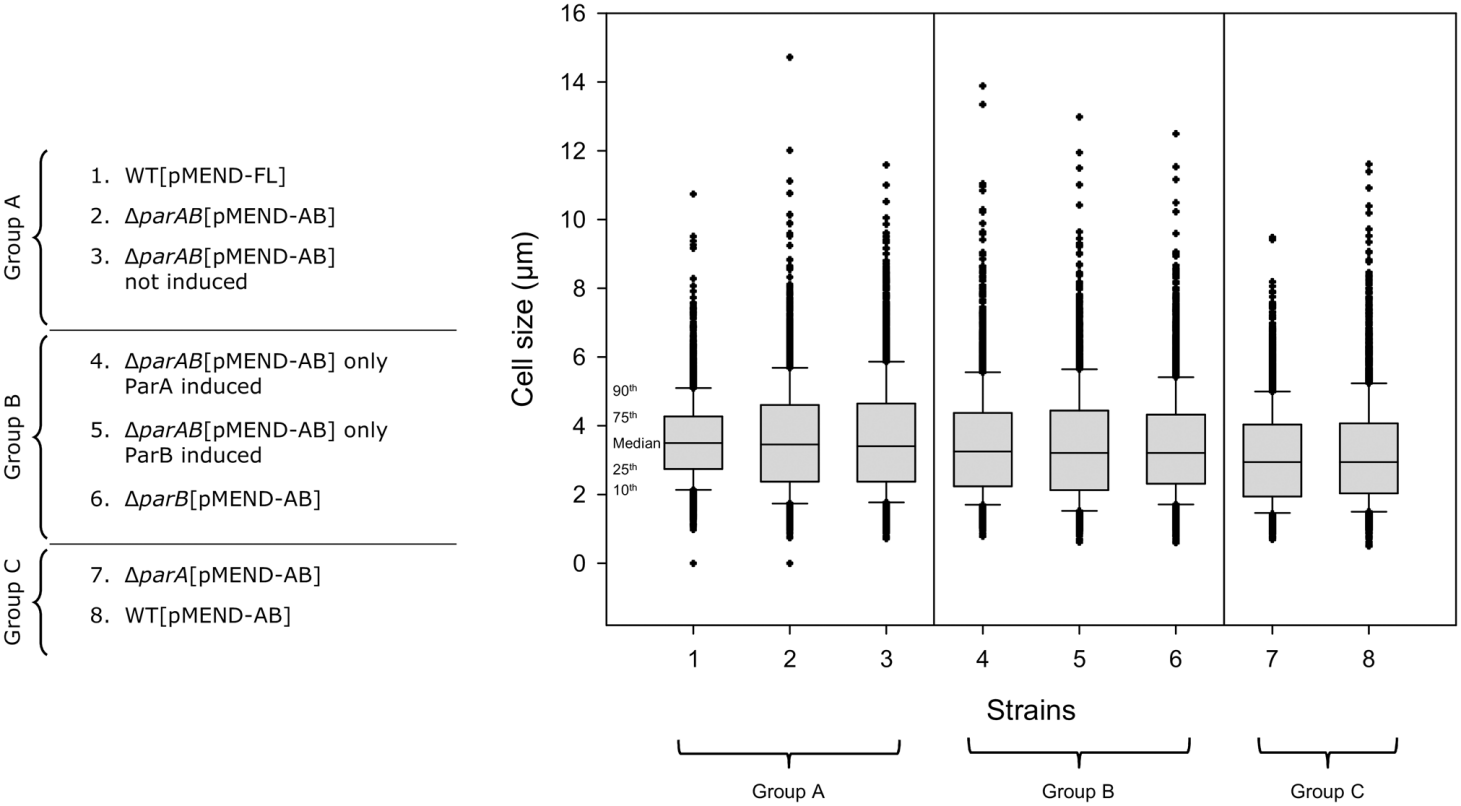
Differences in cell size in *M*. *smegmatis* mc^2^155 cells overproducing ParA-mCherry and/or ParB-EGFP. In the cells harbouring plasmid pMEND-AB, both ParA-mCherry and ParB-EGFP are induced except in the cases indicated. Differences in median cell size values were calculated using the Kruskal-Wallis One Way Analysis of Variance on Ranks. To isolate the groups that differ from the others, All Pairwise Multiple Comparison Procedures (Dunn’s Method) were used. Box plots are shown representing the cell size of each of the strains. From bottom to top, 10^th^ and 25^th^ percentiles, median, 75^th^ and 90^th^ percentiles are plotted. Outliers are indicated by black crosses. The strains are clustered in three different groups (A, B, and C). Within each group, there are non-significant differences in median size between strains. The strains within each group have a statistically significant difference in median cell size from any of the strains represented in either of the two other groups (P<0.05). The median cell size is largest in A>B>C. The number of cells analysed for each strain were: (1) 2,865; (2) 2,812; (3) 2,780; (4) 2,067; (5) 2,654; (6) 2,798; (7) 2,744; and (8) 3,044.

To study the dynamics of ParA-ParB we transformed the integrative plasmid pMEND-AB, which expresses both inducible ParA-mCherry and ParB-EGFP, into wild type (WT), Δ*parA*, Δ*parB*, and Δ*parAB* backgrounds. Growth curves for Δ*parA* expressing ParA-mCherry and Δ*parB* expressing ParB-EGFP showed at least partial complementation indicating the fusion proteins are functional (S1 Fig). However, further analysis illustrated how perturbing this system produced pronounced effects on bacterial growth and chromosome segregation. We chose the Δ*parAB* [pMEND-AB] strain for further study, since it displayed the least pronounced defects: a) differences in median cell size are not statistically significant between WT and Δ*parAB* [pMEND-AB] strain (Fig 1); b) numbers of minicells (≤2.25 μm) in this strain (28%) are similar to those in the WT control (18%) (Table 1); c) although the percentage of anuclear cells in this strain (10.7%) was higher compared to the WT control (1.6%), it was the least perturbed among the different strains (Table 1); d) this is one of the strains closest to WT growth characteristics in both the microfluidic chamber (S1 Table) and batch culture (S2 Fig). Western blots (S3 Fig) of whole cell lysates probed with anti-ParA and anti-ParB antibodies showed no expression in the knock out, and expression of both ParA and ParB in wild-type and complemented cells; there is some evidence of leakiness with the inducible constructs, but high levels of background with the anti-ParA antibody, which we were not able to decrease, make interpretation difficult. The characteristics of the Δ*parAB* [pMEND-AB] strain is a potential limitation of this study, which may impact on ParAB dynamics, although we cannot visualise the behaviour of the native proteins for comparison. During the preparation of this manuscript a paper published by Ginda *et al* [22] reports similar results on ParAB positioning and movement using allelic replacement rather than chromosomal insertion to create reporters.

**Table 1:** Percentage of anuclear cells and minicells (cells ≤2.25 μm in length) in *M*. *smegmatis* mc^2^155 overproducing ParA-mCherry and/or ParB-EGFP.

		Minicells	Anuclear cells		
Genomic background	Plasmid	Number of cells analysed	% anuclear cells	Number of cells analysed	% minicells
WT	pMEND-FL (control)	6310	1.6	2865	18.4
WT	pMEND-A	5547	13.3	2974	35.1
WT	pMEND-B	6478	3.2	2987	19.2
WT	pMEND-AB	6165	12.3	3044	37.3
Δ*parAB*	pMEND-AB	7507	10.7	2812	28
Δ*parAB*	pMEND-AB/ Only ParA-mCherry induced	6394	10.7	2067	31.7
Δ*parAB*	pMEND-AB/ Only ParB-EGFP induced	5655	17.1	2654	34.2
Δ*parAB*	pMEND-AB/ No induction	7026	12.6	2780	28.3
Δ*parA*	pMEND-AB	5241	12.5	2744	39.9
Δ*parB*	pMEND-AB	3835	8.7	2798	30.9

In the cells harbouring plasmid pMEND-AB, both ParA-mCherry and ParB-EGFP are induced for production except where indicated. Anuclear cells were quantified by staining with DAPI as described in Methods.

The dynamics of ParA-mCherry and ParB-EGFP were analysed in 124 cells over 9 hours using time-lapse fluorescence microscopy to observe multiple cell divisions, and track cell lineages. 56% of the cells analysed contained one or two ParB-EGFP foci, while 34% of cells had or produced a total of 3 or 4 ParB-EGFP foci. However, this probably represents two daughter cells prior to the completion of cytokinesis, resulting in a maximum number of ParB foci of 2 per daughter cell. ParB-EGFP spots could not be tracked in 4% of cells, and the remainder appeared to be anuclear (6%).

### Patterns of ParA and ParB localisation

**Fig 2:**
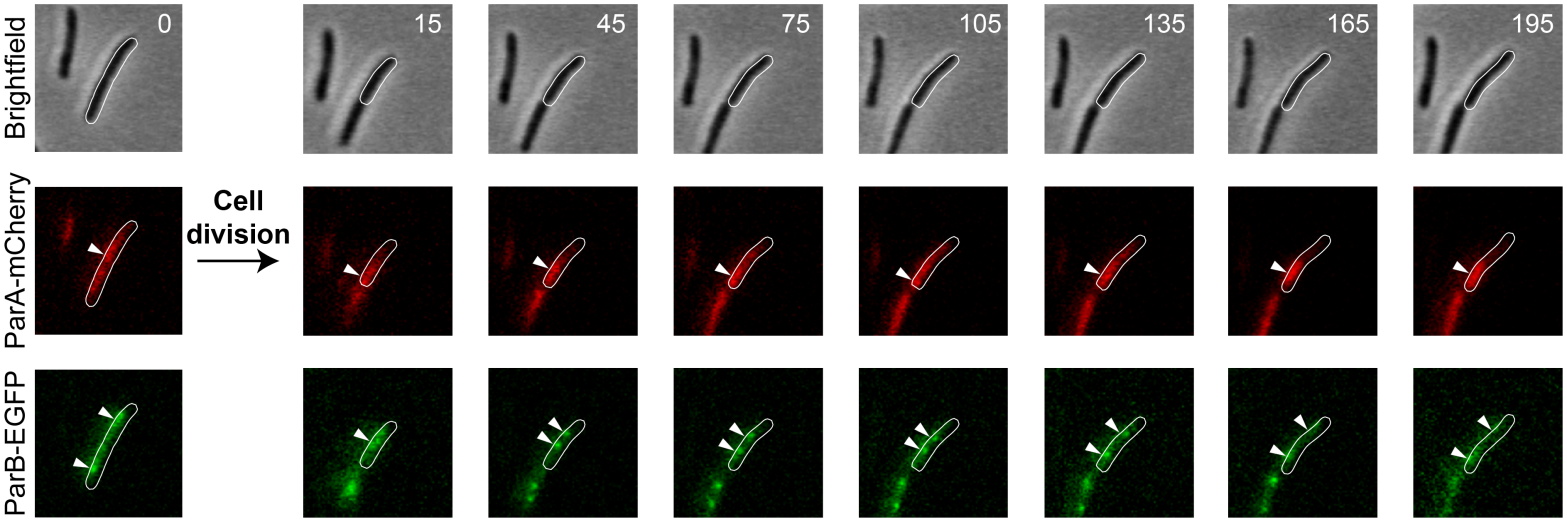
Example of ParA-mCherry and ParB-EGFP dynamics in *M*. *smegmatis* mc^2^155 Δ*parAB* [pMEND-AB]. A selection of images from a 9 h time-lapse experiment is depicted. The dynamics of ParA-mCherry and ParB-EGFP are shown separately. Just before cell division, ParA accumulates in the midcell area, which will become the new pole of the daughter cells. At this point the two ParB foci are situated at symmetrical subpolar positions. After the division of the cell, a ParB focus situated near midcell in the newborn cell splits in two. One of the new ParB foci moves towards the new pole of the cell (where there is a higher concentration of ParA) whereas the other moves towards the old pole. White arrows indicate the area of maximum ParA concentration and the localisation of the ParB foci. Numbers in the top right corner of the pictures indicate time elapsed (in minutes) since the first frame.

We investigated the relationship between ParB movement and local concentrations of ParA. Although we are working with an asynchronous population of cells in which at any given time point we can see cells in all stages of cell growth and division, time-lapse microscopy allows us to artificially synchronise these populations *in silico*. We studied the colocalisation of labelled ParB foci with labelled ParA in single cell lineages. We see accumulation of ParA-mCherry close to the new pole of the cell (the septum area when cells are conjoined). A single ParB focus is usually located near midcell—as already reported in mycobacteria [7,8]—and when it splits one focus moves towards the area of concentrated ParA; the other moves towards the old pole, until they reach near symmetric sub-polar positions (Fig 2).

**Fig 3:**
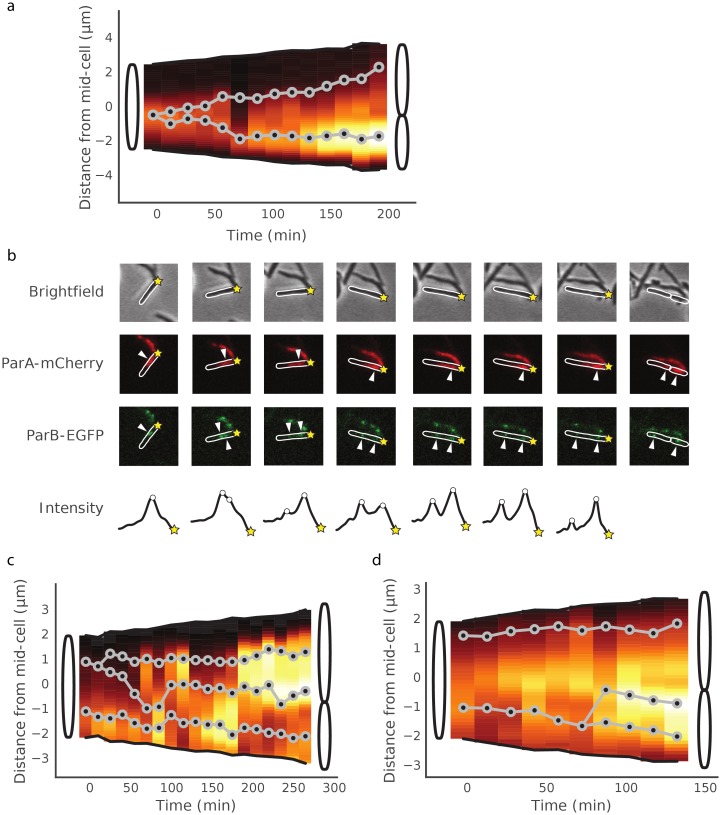
Analysis of ParA-mCherry and ParB-EGFP dynamics in *M*. *smegmatis* mc^2^155 Δ*parAB* [pMEND-AB] single cells. (a, c, d) ParA-mCherry dynamics are represented as a concentration gradient covering the entire cell. Black: minimum ParA concentration; white: maximum ParA concentration. ParB-EGFP foci dynamics are represented as gray lines with black markers. Each line represents the movement of a single ParB-EGFP focus. Cells are drawn such that the new pole of each cell is always situated at the bottom of the graph. (a) One ParB-EGFP focus splits into two foci. This figure represents a single cell in which a single ParB-EGFP focus splits, and one of the foci moves towards the new pole of the cell, towards an area where the ParA concentration is simultaneously increasing, whereas the other focus moves towards the old pole. The cell divides into two daughters at the end of the period shown. A subset of the time-lapse images of the cell represented in cartoon (a) are shown in panel (b). The ParA-mCherry maximum and ParB-EGFP foci are denoted with white triangles. The ParB-EGFP intensity trace of the cell is depicted in the fourth row, with the raw intensity in gray, the smoothed intensity in black, and the assigned ParB-EGFP foci denoted with white circles. The new pole of the cell is denoted by a yellow star. (c, d) Three ParB-EGFP foci per cell. Two independent cells with two ParB-EGFP foci at the start of the visualisation period, in which one of the foci splits and moves towards the midcell area, where the ParA-mCherry concentration is higher. (c) The focus closer to the old pole splits. (d) The focus closer to the new pole splits. Both cells in (c) and (d) divide into two daughters at the end of the period shown.

**Fig 4:**
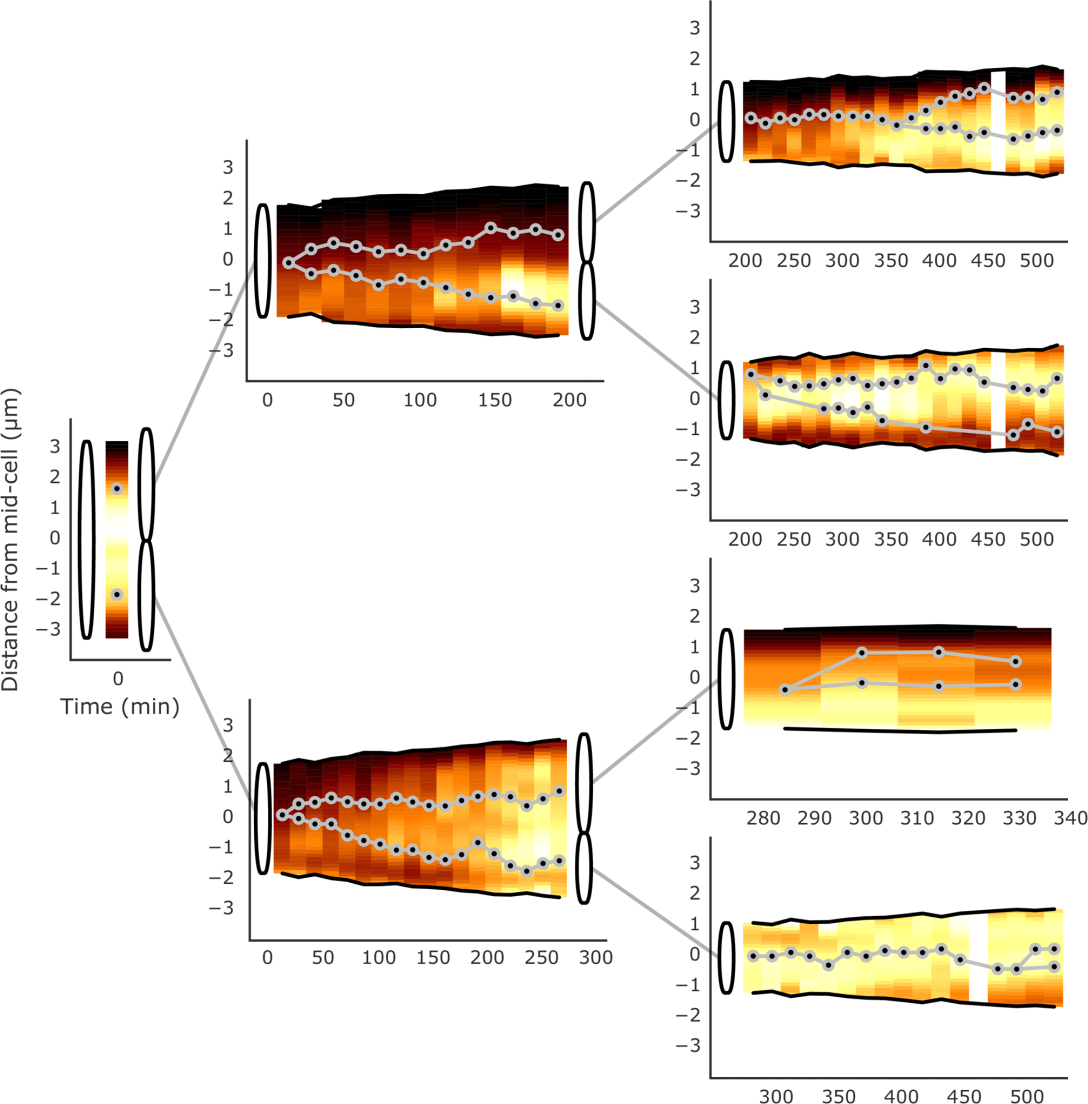
Analysis of ParA-mCherry and ParB-EGFP dynamics in a *M*. *smegmatis* mc^2^155 Δ*parAB* [pMEND-AB] lineage of cells. Two ParB-EGFP foci per cell. Dynamics are depicted as in Fig 3a. This figure represents a lineage of cells starting with a single cell that divides twice to result in four daughter cells. In this lineage, all cells whose birth can be tracked are born with a single ParB-EGFP focus that splits into two.

Fig 3a and 3b shows the analysis of a typical example (69%; n = 48) where ParB-*parS*, located near midcell, replicates and one spot remains with the higher concentration of ParA close to the new pole, while the other spot moves towards the old pole where there is less ParA (this pattern was observed in 78% of cells in which one ParB spot splits into two). We also observed cells in which additional rounds of chromosome replication commence prior to the separation of daughter cells, as previously reported [23]. In the two cases shown in Fig 3c and 3d, we see a cell with two fluorescent ParB-*parS* spots, one of which splits again before cytokinesis, in each case leading to one daughter cell that is born with two *ori*-proximal regions. In 64% of these cases, one focus moves towards midcell, close to where the septum is forming and the concentration of ParA is higher. We were unable to follow these cells further to establish how long this inheritance of two chromosomes continues, or what consequences it might have. We occasionally see lineages where the mother cell produces daughter cells with 2 *ori*-proximal regions in each (S4 Fig). S5 Fig shows an example of analysis of cells starting with two ParB spots that go on to produce daughter cells with one chromosome each; in such cells we have missed the initial ParB duplication event. Fig 4 displays a lineage of cells showing ParAB dynamics over two rounds of cell division.

### The ParA local maximum is inherited by the larger sister cell

**Fig 5:**
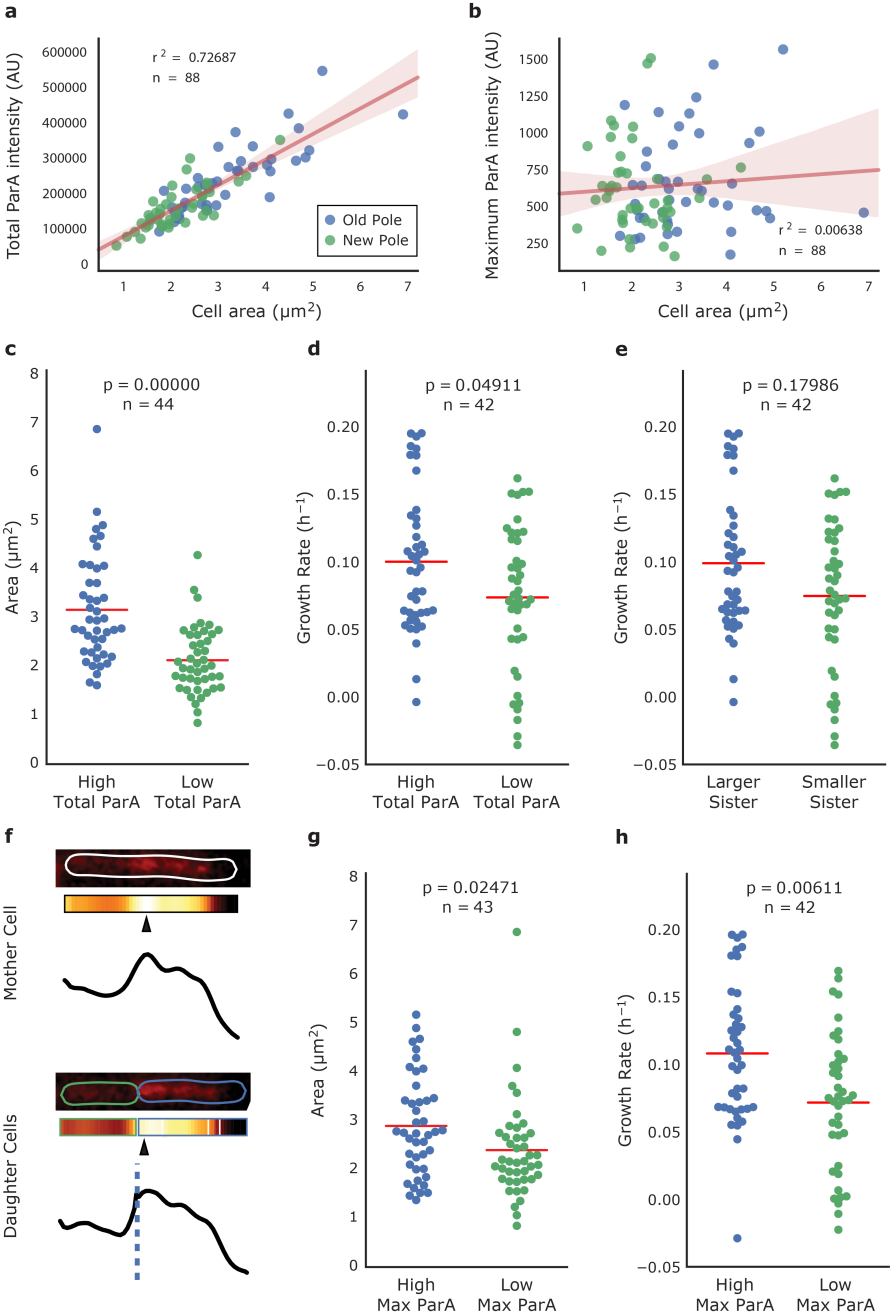
ParA-mCherry inheritance in *M*. *smegmatis* mc^2^155 Δ*parAB* [pMEND-AB]. Whereas there is a direct relationship between cell area and total ParA-mCherry intensity at birth within the population (a), there is no relationship between cell area and the maximum intensity of ParA-mCherry at birth within the population (b). *n* = number of cells analysed; *r*^2^ = coefficient of determination. The least squares linear regression line is depicted as a solid line, and the 95% confidence of this fit is represented by the shaded region. (c-e) Sister cells that inherit a higher level of total ParA-mCherry (high inheritor; blue) have a greater area (c) and grow at a faster rate (d) than low inheritors (green). When analysed independently of ParA-mCherry inheritance, we do not observe a statistically significant difference in growth rate between larger and smaller sibling cells (e). (f) An example cell division where a peak of ParA-mCherry (denoted by filled triangles) is inherited by the larger sister cell (blue). (g, h) Despite a lack of relationship between cell area and maximum ParA-mCherry intensity in the population, sister cells that inherit the local region of maximum ParA-mCherry intensity (high inheritor; blue) are larger (g), and grow at a faster rate (h) than their sisters (low inheritor; green). Mean values are depicted with a red line, and P-values were calculated using Welch’s t-test.

In order to further investigate the dynamics of ParA-mCherry and how it relates to the movement of ParB and the chromosome, we measured ParA-mCherry fluorescence intensity along the length of Δ*parAB* [pMEND-AB] cells at birth, determined total and maximum fluorescence intensity, and looked for relationships between ParA fluorescence and cell area at birth. Fig 5a shows a direct relationship between cell area and total ParA fluorescence as expected by random partitioning of cellular contents, but Fig 5b shows that within the population, there is no relationship between cell area and the maximum intensity of ParA fluorescence. These results indicate that while larger cells have more ParA-mCherry, this is not correlated to the distribution of ParA-mCherry within each individual cell. In Fig 5c–5h this analysis is repeated on sibling pairs of cells to look at how ParA is distributed between sisters. In agreement with the population analysis, as expected by random partitioning, the siblings that inherit more total ParA are significantly larger than their sibling (Fig 5c), and grow significantly faster (Fig 5d). Although ParA maximum intensity is uncorrelated to birth cell area in the population (Fig 5b), when sibling cells are compared, the sibling that inherits a maximum region of ParA fluorescence is significantly more likely to be larger (Fig 5f and 5g; S6 Fig) and grow faster (Fig 5h) than its sibling. We subsequently sought to determine whether this preferential inheritance of the ParA maximum is due to a specific mechanism or if it is random. The probability that a randomly distributed maximum is inherited by a particular daughter cell post-division can be estimated by the ratio of daughter cell size to parent cell size. By summing these ratios over all cell divisions the number of cells that inherit the ParA maximum can be predicted. Looking at 44 cell divisions, the expected number of inherited ParA maxima by the larger sibling cell as predicted purely by cell size is 26.38, and the actual number inherited by the larger sibling is 27. Thus, inheritance of the ParA maximum is consistent with a size-based non-specific mechanism that may be a consequence of asymmetric division itself. As larger sisters do not have a statistically significant increase in growth rate when analysed independently of ParA inheritance (Fig 5e), it raises the possibility that inheritance of the ParA maximum might confer a growth advantage, and it would be interesting to address this in the future. Therefore, while inheritance of the peak region of ParA fluorescence is not linked to a larger cell birth size within the population, it is significantly more likely to be inherited at division by the larger (and faster) sister cell in a sibling pair at division.

### Movement of ParB and the chromosome is an active process

**Fig 6:**
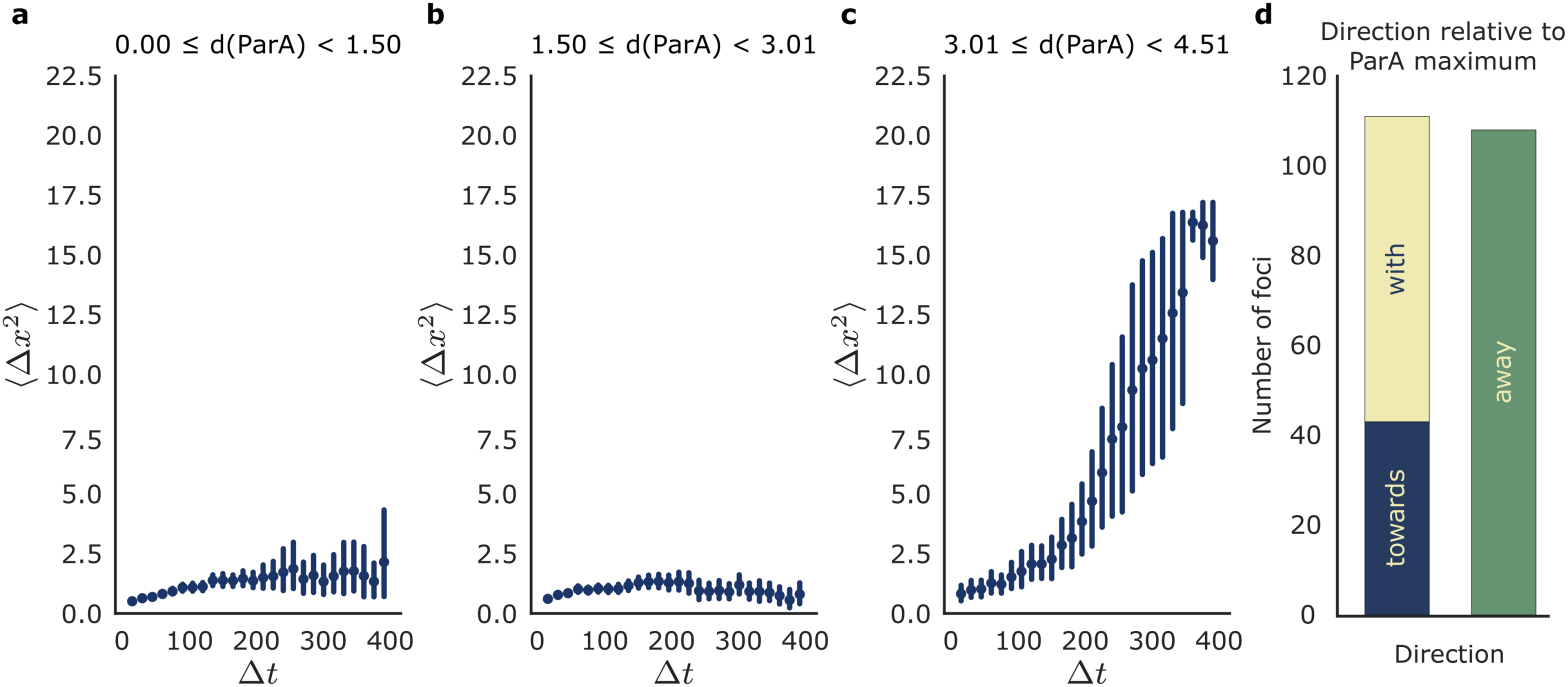
Movement of ParB-EGFP foci in *M*. *smegmatis* mc^2^155 Δ*parAB* [pMEND-AB]. (a-c) Mean squared displacement of 219 ParB-EGFP foci relative to the ParA maximum position over time. For each ParB foci, squared change in distance (Δ*x*^2^) from the ParA maximum over increasing time windows (Δ*t*) is determined, with values binned according to the initial distance of ParB from the ParA maximum at *t* = 0. The mean squared displacement (〈Δ*x*^2^〉) is plotted for each time window (Δ*t*) with error bars showing the 95% confidence interval of each mean. ParB foci originating less than 1.5 μm from the ParA maximum (a) have low mean squared displacement, and are sub-linear, indicating passive and limited sub-diffusion. ParB foci between 1.5 and 3 μm from the ParA maximum (b) retain this sub-diffusive pattern, but ParB foci between 3 and 4.5 μm (c) show a distinct non-linear increase in mean squared displacement with time, indicating non-passive movement. The direction of the movement of ParB-EGFP foci was measured in relation to the ParA maximum with foci classified as moving toward, with, or away from the maximum according to their relative velocity, and the numbers of each type of movement are depicted in (d).

Our analysis of the dynamics of ParB-EGFP in *M*. *smegmatis* Δ*parAB* [pMEND-AB] indicates that active processes might contribute to chromosome movement during cell growth. To assess whether ParB is moving actively, we determined the diffusion profile of ParB-EGFP foci relative to the maximum region of ParA-mCherry (219 ParB-EGFP spots in 124 cells were analysed). Diffusion can be examined by fitting mean squared displacement from an initial position as a function of elapsed time (Δ*t*). The shape of this relationship distinguishes between various types of diffusion, using the equation: 〈Δ*x*^2^〉 = 2*D*. (Δ*t*)^*β*^. A *β* of 1 (i.e. a linear relationship) implies pure passive diffusion, a *β* of less than 1 implies anomalous diffusion or sub-diffusion and a *β* greater than 1 implies active movement [24]. Mean squared displacement for ParB foci that originate close to ParA (Fig 6a) is low, indicating limited movement when ParB is near ParA, with *β* = 0.4 implying sub-diffusive movement, which is consistent with a molecule that cannot diffuse freely. This regime is maintained for ParB foci at an intermediate distance from the ParA maximal region (Fig 6b), with very limited movement. However, ParB foci that are far from ParA (Fig 6c) clearly move in a non-linear manner, with greatly increasing mean squared displacement over time. This is a clear indication that ParB movement is non-passive (active) when >3 μm from a maximum region of ParA-mCherry. We also determined the directionality of ParB foci, and observed foci that move towards the area of maximum ParA concentration, maintain their distance, or move away. 51% of ParB foci move towards or stay close to the maximum ParA area, whereas the other 49% move away from it (Fig 6d), as expected for correct chromosome segregation.

### Chromosome segregation defects produce anuclear cells and minicells

We induced ParA-mCherry, ParB-EGFP, or both proteins in WT and different mutant background strains (Δ*parA*, Δ*parB*, and Δ*parAB*) to investigate the effect that altering protein expression has on cell growth and division. For this, we used the integrative plasmids pMEND-AB, pMEND-A, and pMEND-B, in which *parA-mcherry* and *parB-egfp* are under the control of inducible promoters. We counted the number of anuclear cells and minicells as indicators of chromosome segregation abnormalities. We determined the number of anuclear cells by DAPI staining and measured cell size in the different strains. WT harbouring plasmid pMEND-FL (see Methods) was used as a control.

In a WT background induction of ParA and ParB together, or ParA alone, produces higher numbers of anuclear cells than induction of ParB alone (Table 1). Interestingly, removing the chromosomal copy of *parB* and expressing both proteins from pMEND-AB reduces the number of anuclear cells compared to WT [pMEND-AB] strain, suggesting that deleting *parB* and therefore reducing ParB expression, partially alleviates the missegregation defects (Table 1). These data support the hypothesis that when native ParA levels are altered (increased or decreased) chromosomal segregation via ParB (at native or increased levels) is severely altered. These defects seem to be milder if ParB levels are lowered. However, when native levels of ParA protein are present, chromosome segregation seems less affected by excess ParB (WT [pMEND-B]), with fewer anuclear cells and minicells (Table 1). Induction of ParB in a Δ*parAB* mutant produces the highest number of anuclear cells (Table 1), which agrees with previous work showing that a *parA* null mutant has a more marked phenotype than either a *parB* mutant or a *parAB* double mutant [6].

The strains with the highest numbers (>37%) of minicells are Δ*parA* [pMEND-AB] and WT [pMEND-AB] (Table 1), which have a statistically significant shorter median cell size compared to the others (Fig 1). This suggests that altered ParA levels impact cell size more than the complete absence of ParA, which in turn would have more impact on the development of anuclear cells. These results underscore the importance of balancing levels of ParA and ParB proteins for maintaining cell size, with native levels of ParA able to alleviate the effect of increased levels of ParB.

## Discussion

**Fig 7:**
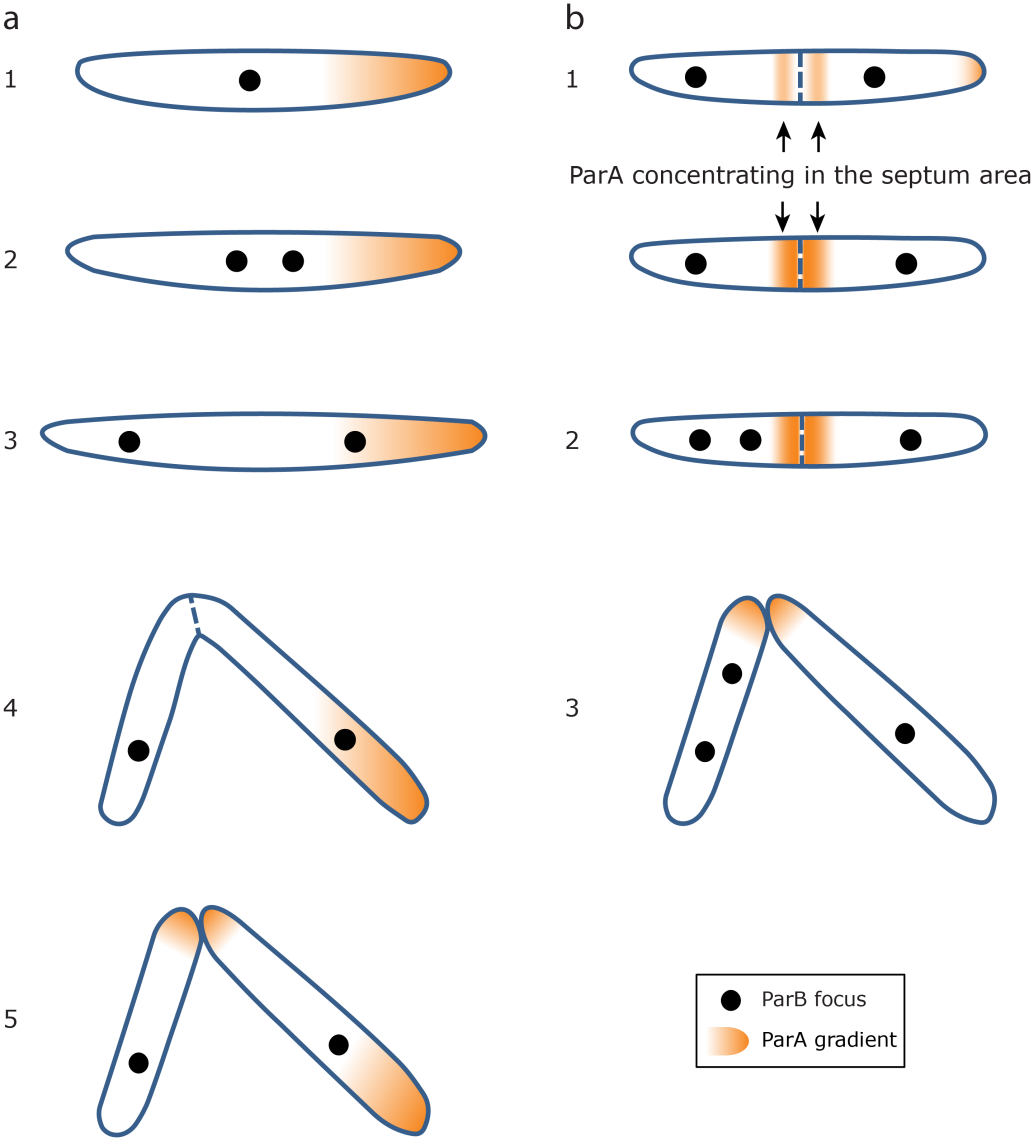
Model of ParA and ParB dynamics. (a) Model describing dynamics in an individual (non-conjoined) cell 1: A single ParB focus is situated usually at midcell (it can also be shifted towards one of the poles). 2: The ParB focus splits into two. One of the new foci moves towards or stays close to the region with the highest concentration of ParA (situated near the new pole in at least 69% of cells). 3: The ParB focus near the old pole moves towards it, perhaps triggered by DNA-cell envelope interactions. 4: The septum is formed and the two daughter cells divide. 5: ParA accumulates at the new poles created by the septum. A new round of segregation can begin. (b) Model describing dynamics in conjoined cells. 1: In a cell already containing two ParB foci, the ParA gradient situated near the new pole moves or stays close to the area of septum formation (that will become the new pole of the daughter cells). 2: One of the ParB foci splits and one of the new foci moves towards/stays close to the septum area whereas the other new focus moves towards the pole. In the figure, only the conjoined sister cell on the left experiences this second round of segregation, but both conjoined sisters can undergo this second round of replication (resulting in 4 ParB foci before cytokinesis). 3: The sister cells split (one inherits two ParB foci).

We report here a detailed analysis of the movement of both ParA and ParB during the growth and division of *M*. *smegmatis* using time-lapse microscopy and microfluidics, and propose a model of their dynamics (Fig 7). As described for other microorganisms, ParA forms concentration gradients that accumulate near the new pole (Fig 7a) or close to midcell in conjoined cells where the new pole is forming (Fig 7b). ParB localises to *parS*-sites on the chromosome near *oriC*. Once *oriC/parS* replicates, two spots appear, and one of the newly replicated chromosomes moves towards or remains close to the highest concentration of ParA, whereas the other moves towards the old pole. These observations indicate that the localisation of two ParA foci near the cell poles previously reported [6] is probably an artefact of the non-growing cells used, where the lack of ATP might cause ParA to associate with the poles by default. During preparation of this manuscript, an article was published also describing the dynamics of ParA and ParB in mycobacteria [22]. This replicates many of our results using an alternative allelic replacement approach, and proposes a model of ParAB dynamics almost identical to that in Fig 7. However, we present additional novel data about ParA and ParB behaviour that complements the results published by Ginda *et al* [22]. We observed that the concentration of fluorescent ParA in a cell is directly proportional to cell size at birth, so that all cells start with a similar ParA concentration (Fig 5a). However, ParA is not uniformly distributed within the cell; a region of maximum intensity can be identified, which is not correlated with cell area at birth at the population level. Nevertheless, this concentrated region is preferentially inherited by the larger daughter cell when sibling pairs are analysed (Fig 5f and 5g). We do not observe a significant increase in growth rates in larger sister cells in our data (Fig 5e), although they do inherit a concentrated region of ParA; whether or not this confers a growth advantage, or if there is a causal link between growth rate and ParA inheritance is unknown. This is the first time differential inheritance of ParA has been described in mycobacteria.

Our observations on live cells support a model where ParA initially localises near the new pole of the cell, as described recently [22]. However it has also been reported [6] that ParA interacts with the polar determinant Wag31, a protein that preferentially localises at the old pole of the cell [25]. It may be that Wag31 interactions are transitory, and other as yet unknown proteins are involved in ParA recruitment to the new pole, perhaps in a similar fashion to the TipN protein in *C*. *crescentus* [13,14].

Active movement of ParB was another surprising and novel finding of this study. When ParB is close to an area of high ParA concentration, it displays sub-diffusive movement, which suggests that protein-protein interactions with ParA restrain ParB movement. When ParB is farther away from ParA, it shows active movement, either towards the ParA-concentrated area or towards the opposite pole (usually the old pole). This implies that ParB segregation—and consequently chromosome segregation—is, at least in part, an active process. The movement towards the old pole might be triggered by the chromosome binding to the cell envelope near it, using a mechanism similar to the chromosome-anchoring proteins described for *Caulobacter* (ParB-PopZ) and *Bacillus subtilis* (RacA-DivIVA) [26], although such proteins remain unidentified in mycobacteria.

We find the ParB-EGFP focus prior to splitting is usually localised close to midcell. In agreement with our results, other authors have reported that single ParB-reporter foci produced from its native promoter by allelic replacement, appear at midcell and slightly shifted towards the old pole [7,8].

In some cases, in cells with two ParB foci, a second round of chromosome replication initiates before sister cells have completely separated (Fig 7b). In this case, ParA concentrates near midcell, close to where the septum (and therefore the future new pole) is forming. One or both ParB foci then split and move towards/stay close to midcell. There seem to be two different ParA gradients that act independently in each conjoined sister cell. This would support the suggestion [23] that sister cells start functioning as independent metabolic entities while still physically joined. Whether the ParA gradient plays a role in septum positioning [6] remains unclear. We observed fewer cells (34%) starting a second round of chromosomal replication before cell separation than others have reported (78% [23]), which may be due to the different experimental systems used. In contrast to the Santi study [23], where the chromosomes in both conjoined sisters replicated, in our analysis the segregation of ParB (and therefore the chromosome) in conjoined sisters mainly proceeded in an asymmetric fashion (one daughter duplicates the chromosome before division; the other does not) however, the defects in growth and segregation seen of the Δ*parAB* [pMEND-AB] strain may bias the results. Therefore, further analysis is required to determine if this is a consequence of the relative levels of ParB in the recombinant strain used [7,8]. However, we have observed that the ParAB dynamics at the single cell level seem not to be affected by this asymmetric chromosome segregation in conjoined cells, being almost identical to that described in a WT strain expressing reporter-fusions from *parA* and *parB* in their native loci [22].

There are various models of ParA/ParB dynamics [14,16–18], and while our study does not provide conclusive evidence for any specific mechanism, our observations do constrain future mechanistic models of ParA/ParB dynamics. For example, we conclude that the dynamics of ParB foci imply active transport when they are located at certain distances away from the highest concentration of ParA. A realistic mechanistic model should also explain why growth and chromosome segregation is more sensitive to perturbations in the levels of ParA rather than ParB. Our study of strains with differing ParA and ParB gene copy number confirms the previous conclusion [3,6] that a correct balance of these proteins is important for accurate segregation of the chromosomes. However, we also demonstrate that it is more important to maintain optimal levels of ParA rather than ParB, as missegregation errors are more evident not only when ParB is present in the absence of ParA, but also when ParA levels are reduced or increased. Indeed, increased ParB does not produce major growth errors if native levels of ParA are present. ParA might only be able to interact correctly with ParB-*parS* complexes within a relatively narrow range, and outside this range ParB behaviour might be the main cause of missegregation errors.

The relationship between growth rate and pole age has been addressed in several studies that reach different conclusions as to whether cell growth is exclusively unipolar (from the old pole) [27] or bipolar [23,28,29]. In future it would be interesting to analyse a putative relationship between pole age and inheritance of the ParA concentration maximum.

## Methods

### DNA manipulation, bacterial strains and growth conditions

The strains and the plasmids used in this work are listed in S2 Table. Primers used in this work are listed in S3 Table. *Mycobacterium smegmatis* mc^2^155 [30] and derivatives were grown in defined liquid medium, either Middlebrook 7H9 (OADC supplemented, Difco) or Hartmans-de Bont minimal medium [31], both supplemented with 0.05% Tween-80. Liquid cultures were grown aerobically at 37°C in an orbital shaker at 180 rpm. For growth on solid medium, mycobacteria were grown on Middlebrook 7H11 agar (OADC supplemented, Difco) incubated at 37°C. When needed, hygromycin (50 μg/ml) and kanamycin (20 μg/ml) were used for plasmid selection and maintenance. Tetracycline (5 ng/ml) and theophylline (2 mM; Sigma) were used to induce mCherry and EGFP respectively. *Escherichia coli* DH5α was used as a host for cloning. It was grown in LB medium at 37°C in an orbital shaker at 180 rpm. LB agar plates were used. Ampicillin (25 μg/ml), hygromycin (150 μg/ml) and kanamycin (50 μg/ml) were used for plasmid selection and maintenance.

### Construction of *M*. *smegmatis* mutants

For the disruption of *parA*, the pNIL/GOAL method of making marked mutations in mycobacteria was used [32]. An NcoI site near the 5’ end of *parA* was chosen as the site for disruption. A region of approximately 2.4 kbp was selected centred on the NcoI site and amplified. The resulting DNA fragment was cloned into the HindIII site of p2NIL, creating p2NIL-parADel. The hygromycin cassette from pSE100 was amplified and cloned into the NcoI site within p2NIL-parADel creating p2NIL-parADel-HygR. Finally, the gene marker cassette from pGOAL17 encoding the genes *sacB* and *lacZ* was cloned into the PacI site of p2NIL-parADel-HygR to create the suicide vector pAEV-parA to disrupt *parA*. Electrocompetent *M*. *smegmatis* mc^2^155 cells were transformed with pAEV-parA and single crossovers selected on plates containing hygromycin and kanamycin. A single colony was selected and grown in broth without antibiotics to allow a further crossover event to take place, and double crossover mutants were selected on plates containing hygromycin, 10% sucrose, and X-gal (5-bromo-4-chloro-3-indolyl-β-D-galactopyranoside). White colonies should represent double crossover mutants (loss of the *lacZ* gene marker cassette but retention of hygromycin resistance) and were replica plated onto kanamycin plates and hygromycin plates to confirm the loss of the kanamycin resistance cassette located on pAEV-parA. *parA* disruption was confirmed by PCR and Southern blotting.

The unmarked *M*. *smegmatis* Δ*parB* mutant was kindly provided by Dagmara Jakimowicz [1]. To produce a Δ*parAB* double mutant, mycobacteriophage driven homologous recombination was used [33]. Electrocompetent *M*. *smegmatis* Δ*parB* cells were transformed with plasmid pJV53 [34], which facilitates double-stranded DNA recombination. Subsequently, acetamide-induced *M*. *smegmatis* Δ*parB* pJV53 electrocompetent cells were transformed with the 3.7 kbp linear XmnI/KpnI fragment excised from p2NIL-parADel-HygR; this contains *parA* sequences flanking the hygromycin cassette. Transformants were selected on hygromycin plates and confirmed by PCR and Southern blotting. To remove the pJV53 helper plasmid the Δ*parAB* mutant was grown in the presence of hygromycin for four generations and colonies were replicated onto plates containing hygromycin plus kanamycin or hygromycin alone. A colony that only grew on hygromycin plates was selected as having lost the kanamycin resistant helper plasmid.

### Construction of plasmids

pMEND-A, an integrative plasmid harbouring a *parA-mcherry* fusion under the control of a tetracycline-inducible promoter was constructed by cloning the PCR-amplified *MSMEG_6939* (*parA*) into the BamHI—NdeI sites of pMEND-mCherry (ensuring an in-frame fusion). A RBS was included in the 5’ primer 7 bp upstream of the *parA* start codon, and the integrase cassette from pMEND-int [35] was subcloned as a AgeI—MfeI fragment into pMEND-ParA-mCherry to produce the integrative version.

pST-B contains a *parB-egfp* fusion under the control of a theophylline inducible promoter, and was constructed by cloning the PCR-amplified *MSMEG_6938* gene (*parB*) (ensuring an in-frame fusion to the N-terminus of EGFP) into the EcoRI site of the episomal plasmid pST5552 [36].

pMEND-B is an integrated, single-copy, theophylline-inducible ParB-EGFP, constructed by PCR amplifying 2331 bp containing the *riboswitch-parB-egfp* fragment from pST-B and cloning it into the PacI site of pMEND-mCherry-int.

pMEND-AB, harbouring both *parA-mcherry* and *parB-egfp* under the control of tetracycline and theophylline inducible promoters respectively, was constructed by cloning the same *riboswitch-parB-egfp* fragment into the PacI site of pMEND-A.

pMEND-FL, harbouring *mcherry* and *egfp* genes under the control of tetracycline and theophylline inducible promoters respectively, was used as a negative control plasmid. It was constructed by PCR amplifying 1278 bp containing the *riboswitch-egfp* fragment from pST5552 and cloning it into the PacI site of pMEND-mCherry-int.

Plasmids were electroporated into competent *M*. *smegmatis* mc^2^155 as described previously [37].

### Microscopy and data analysis

Microscopy was performed in the Facility for Imaging by Light Microscopy (FILM) at Imperial College London. Time-lapse live-cell microscopy was performed in B04A plates with a CellASIC^®^ ONIX microfluidic platform (Merck-Millipore). Cells were loaded in the chamber at an OD_600_ of 0.1 from mid-exponential cultures in Hartmans-de Bont medium, and the same medium was flowed at a continuous pressure (1 psi) in a temperature-controlled chamber at 37°C. Fluorescent fusion proteins were induced in cells at mid-exponential phase (OD_600_ of 0.8) with either theophylline (for EGFP; 2 mM) for 5 hours at room temperature in standing cultures, or tetracycline (for mCherry; 5 ng/ml) for 3 hours at 37°C with shaking, before loading the cells in the microfluidic chamber, where they continued to be perfused with the inducer. When induction of both EGFP and mCherry was required in dual reporter strains, induction was done sequentially, starting with theophylline, before loading the cells in the chamber, where they were perfused with both inducers.

Images were captured every 15 minutes using a Zeiss Axiovert 200 inverted widefield microscope fitted with an EM-CCD (C9100-02) camera (Hamamatsu) controlled by HCImage software using a 63X objective. Z-stacks were collected at 1 μm intervals. Images from four independent experiments were analysed using Fiji image processing software [38] to select focused z-slices and to generate time-lapse sequences/movies in an appropriate file format, followed by the semi-automated detection of cell boundaries by MicrobeTracker, a MATLAB software package that detects bacterial cells and describes them using a two-dimensional mesh by splitting the cell into segments perpendicular to the long axis of the cell [39]. Single-cells were arranged into cell lineages and analysed using an automated set of custom-made Python scripts with manual correction of assignment errors. Several general statistics were determined from assigned lineages including: (a) cell length, defined as the length of the central line drawn between cell poles along the cell axis; (b) growth rate, determined by the slope of the line fitted by ordinary least squares linear regression to the logarithm of cell lengths against time; (c) doubling time, defined as the time from cell birth until clear division as marked by snapping or cell wall invagination; and (d) cell polarity, which defines new and old poles for newly born cells.

Fluorescence signals were analysed using an additional set of custom-made Python scripts which performed a series of steps: (1) the fluorescence signal for an image was smoothed using a two-dimensional Gaussian filter using a kernel with a standard deviation of two pixels; and (2) the smoothed signal was mapped using the segments derived from MicrobeTracker to the cell long axis by taking the mean intensity across the width of the cell for each position along the axis.

ParA was visualised as a heatmap along the cell body, with intensity normalised to the maximum ParA intensity across the whole cell lifetime (between birth and division). ParA maximum was defined as the position along the cell long axis with maximum fluorescence intensity.

The ParB signal was further processed to determine the position of fluorescent foci: (1) peaks in ParB signal were detected by the PeakUtils package (https://pypi.python.org/pypi/PeakUtils) which takes the first derivative of the amplitude data and determines where the slope changes from negative to positive, whilst ensuring that peaks are separated by more than 5 pixels, and above a threshold value; (2) the number of false positives were restricted by applying random normally-distributed noise (μ = 0; σ = standard deviation of fluorescence/2) to the smoothed fluorescence trace, re-smoothing using a Gaussian filter, and repeating the peak detection. Peaks that were consistently returned in at least 10 of 20 randomised traces were retained for subsequent analysis; (3) ParB peaks were manually corrected based on comparison of intensity traces and captured images; and (4) ParB peaks were temporally connected into contiguous lines in a semi-automated manner: foci that were present in a subsequent frame in a position 5 pixels or less (relative to a cell pole) away from a focus in a previous frame were connected as a single focus. This approach was extensively manually curated to connect foci that were clearly related. Divisions in ParB foci were all assigned manually and spot ‘siblings’ were assigned based on which foci were closer to a parent spot.

For the analysis of ParB diffusion, the distance between ParB foci and the maximum intensity of ParA was determined at all time points, and changes in distance (displacement; Δ*x*) for all time window combinations along the profile were determined. The foci were binned according to how far they were from ParA when they were first observed (bins were 0–1.5 μm, 1.5–3 μm, and 3–4.5 μm; comparisons for ParB foci at >4.5 μm were discarded due to low numbers), and mean squared displacement (〈Δ*x*^2^〉) for each time window (Δ*t*) calculated for all ParB foci within that bin. The specific shape of the dependence of mean squared displacement on time is indicative of particular types of diffusion. If all diffusion profiles are fitted to the equation 〈Δ*x*^2^〉 = 2*D*. (Δ*t*)^*β*^ where *D* is the diffusion coefficient, and *β* describes the model: *β* = 1 (i.e. a linear relationship) indicates free diffusion, *β* < 1 indicates sub-diffusion, and 1 < *β* ≤ 2 indicates active movement.

Measurement of ParB movement relative to ParA was conducted by determining the distance between each ParB focus and the ParA maximum (as defined above). The velocity of movement relative to ParA was determined by calculating the slope of the linear regression line fitted to these distances over time. A threshold value of 0.15 μm h^-1^ was used, with ParB foci classed as moving *towards*, *away*, or *with* the ParA maximum.

For determining ParA inheritance, analysis was restricted to cells in which at least one cell division event could be observed, allowing sister cell pairs to be established. For each sister cell pair, the following attributes were determined: (a) total ParA intensity, calculated as the sum intensity of all pixels within the cell boundary; (b) maximum ParA intensity, calculated as the maximum intensity of all pixels within the cell boundary; (c) cell length and area; and (d) growth rate, calculated as above. For statistical comparisons between groups, Wilcoxon’s signed-rank test was used, which is a non-parametric test used to compare samples that are related which does not assume normality of the underlying data.

For quantification of anuclear cells, mid-exponential cultures in Hartmans-de Bont medium were washed with phosphate buffered saline (PBS; Sigma), stained with 5 μg/ml DAPI (4',6-diamidino-2-phenylindole, dilactate; Invitrogen) in PBS for 5 minutes at room temperature, washed with PBS and mounted on slides using Mowiol 4–88 (Calbiochem).

Differences in cell size between strains were calculated by the Kruskal-Wallis One Way Analysis of Variance on Ranks [40]. This is a rank-based non-parametric test used to determine if there are statistically significant differences between groups of an independent variable (in this case, the median size of each strain) on a continuous or ordinal dependent variable (in this case, cell size, a continuous variable) without assuming homoscedasticity. We subsequently applied Dunn’s test [41], a non-parametric pairwise multiple comparisons procedure based on rank sums, to isolate the groups that differ from others in the median size. Kruskal-Wallis One Way Analysis of Variance on Ranks and All Pairwise Multiple Comparison Procedures (Dunn’s Method) were applied using SigmaPlot (Systat Software, San Jose, CA).

### Code availability

Custom Python scripts written to analyse microscopy data are freely accessible online. For assigning lineages and calculating cell statistics from MicrobeTracker output, scripts are available at https://github.com/mountainpenguin/lineage. For assigning and tracking ParA and ParB foci, scripts are available at https://github.com/mountainpenguin/spot_analysis.

### Preparation of mycobacterial whole cell lysates and western blots

*M*.*smegmatis* strains were grown in Hartmans-de Bont liquid media to an OD of 0.8 and induced sequentially with theophylline (2mM) and tetracycline (5ng/ml). Acetamide-induced ParAB (a gift from Dagmara Jakimowicz) was included as a control. Bacterial cells following induction were harvested by centrifugation at 3000rpm for 10 minutes. The cell pellet was washed and re-suspended in PBS and complete protease inhibitors (Roche diagnostics), followed by rupture in a ribolyser using 0.1mm silica beads (MP Biomedicals). The lysate was recovered after centrifugation at 13,000rpm for 30 minutes. Total protein concentration was determined using the Pierce BCA assay kit (Thermo Fisher Scientific) and 20μg whole cell lysates were boiled and separated on a 12% polyacrylamide gel (Thermo Fisher Scientific) and transferred onto a nitrocellulose membrane (GE healthcare). The membrane was blocked in TBST (0.05% Tween 20) containing 3% BSA (Roche diagnostics). The membrane was probed with 1:2000 rabbit polyclonal anti-par A (absorbed against *M*. *smegmatis* Δ*parAB*) and affinity-purified anti-par B antibodies (gifts from Dagmara Jakimowicz) overnight at 4°C. The membrane was subsequently incubated with 1:5000 goat anti-rabbit IgG antibody (Thermo Fisher Scientific) for 1 hour at room temperature and developed using the SuperSignal West Femto kit (Thermo Fisher Scientific).

## Supporting information

S1 Fig Growth curves of *M*. *smegmatis* Δ*parA* complemented with ParA-mCherry and *M*. *smegmatis* Δ*parB* complemented with ParB-EGFP. Complemented strains are compared to the WT strain and the non-complemented mutant, both harbouring control plasmid pMEND-FL. Strains were grown in Hartmans-de Bont medium and induced for the production of ParA-mCherry and ParB-EGFP. Growth curves were made with data collected from 3 biological replicates. Error bars indicate standard error of the mean.
(PDF)

S2 Fig Growth of different *M*. *smegmatis* strains harbouring pMEND-AB plasmid. Strains were grown in Hartmans-de Bont medium and induced for the production of ParA-mCherry and ParB-EGFP. Growth curves were made with data collected from 3 biological replicates. Error bars indicate standard error of the mean.
(PDF)

S3 Fig Western blots of whole cell lysates of wild-type, mutant and recombinant *M*. *smegmatis* probed with anti-ParA antibody (Panel A) and anti-ParB antibody (Panel B). Cells were grown in the presence or absence of inducer. **Panel A** (1) wild-type, no inducer; (2) wild-type, plus inducer; (3) Δ*parAB* mutant, no inducer; (4) Δ*parAB* mutant, plus inducer; (5) Δ*parAB* [pMEND-AB), no inducer; (6) Δ*parAB* [pMEND-AB), plus inducer. ParA and ParA-mCherry bands are labelled with white arrows in wild-type and complemented strains. **Panel B** (1) wild-type, no inducer; (2) wild-type, plus inducer; (3) Δ*parAB* mutant, no inducer; (4) Δ*parAB* mutant, plus inducer; (5) Δ*parAB* [pMEND-AB], no inducer; (6) Δ*parAB* [pMEND-AB], plus inducer, (7) acetamide-induced ParB.
(PDF)

S4 Fig Analysis of ParA-mCherry and ParB-EGFP dynamics in a *M*. *smegmatis* mc^2^155 Δ*parAB* [pMEND-AB] lineage of cells. Four ParB foci per cell. Dynamics are depicted as in Fig 3a. This figure represents a lineage of cells starting with a single cell which harbours two ParB-EGFP foci which each split into two foci before the excision of the cell into two daughter cells. In the upper daughter cell, one of the foci subsequently splits into two.
(PDF)

S5 Fig Analysis of ParA-mCherry and ParB-EGFP dynamics in *M*. *smegmatis* mc^2^155 Δ*parAB* [pMEND-AB] single cells. Two ParB-EGFP focus per cell. Dynamics are depicted as in Fig 3a. The new pole in the cell in panel (a) is unknown and this is indicated by both poles coloured in red. The new pole of the cell in panel (b) is situated at the bottom. This figure represents two independent cells in which ParB-EGFP foci have already split at the start of the visualisation period. Both cells divide into two daughters at the end of the period shown.
(PDF)

S6 Fig Distribution of ParA pre- and post-division. 10 cell divisions chosen at random are shown. The top row depicts the mother cell just before division, outlined in red. The second row shows the intensity profile along the cell axis for each mother cell. The third row shows the daughter cells post-division, outlined in blue and red. The bottom row shows the intensity profile for each of the daughter cells, with the division site shown as a blue dashed line.
(PDF)

S1 Table Single cell doubling time, growth rate, and division length of *M*. *smegmatis* mc^2^155 WT, WT [pMEND-AB], and Δ*parAB* [pMEND-AB] in the microfluidic chamber. The values are defined and were measured as described in Methods. Mean values are represented ± the standard error of the mean. *n* = number of cells analysed to calculate each value. All strains were induced for the production of ParB-EGFP and ParA-mCherry.
(PDF)

S2 Table Bacterial strains and plasmids used in this study. (PDF)

S3 Table Primers used in this study. Restriction sites are underlined.
(PDF)

S1 Movie ParA-mCherry and ParB-EGFP dynamics in *M*. *smegmatis* Δ*parAB* [pMENDAB]. Time-lapse video of ParA-mCherry and ParB-EGFP dynamics over an 8 h 45 min period. Images were captured at 15 minute intervals. A selection of the frames from this movie are shown in Fig 1.
(AVI)
