# Supplementary figures and images for: Analysis of ParAB dynamics in mycobacteria shows active movement of ParB and differential inheritance of ParA
Source: PLoS One. 2018 Jun 19;13(6):e0199316. doi: 10.1371/journal.pone.0199316 (PMC6007833; doi:10.1371/journal.pone.0199316)

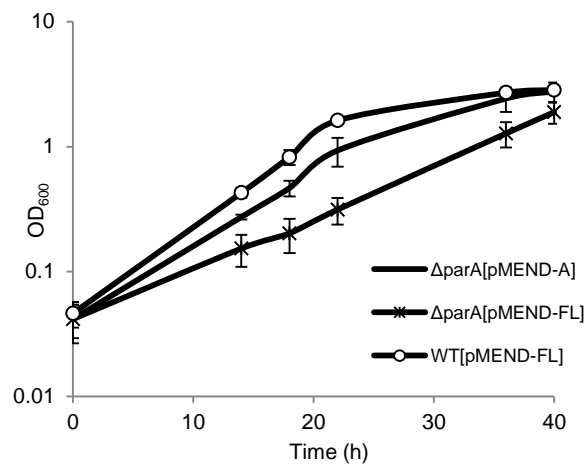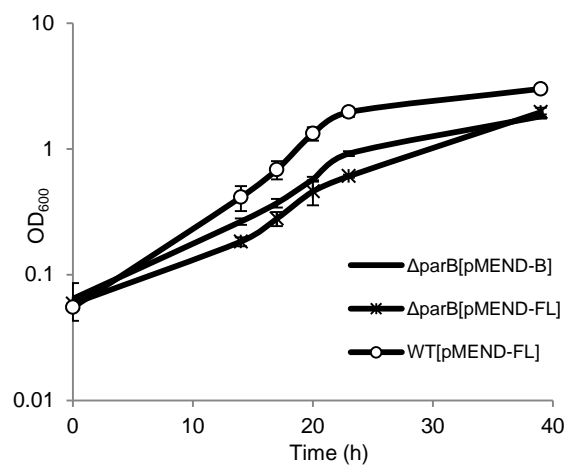

Supplement: S1 Fig — Complemented strains are compared to the WT strain and the non-complemented mutant, both harbouring control plasmid pMEND-FL. Strains were grown in Hartmans-de Bont medium and induced for the production of ParA-mCherry and ParB-EGFP. Growth curves were made with data collected from 3 biological replicates. Error bars indicate standard error of the mean. (PDF) [file pone.0199316.s001.pdf]

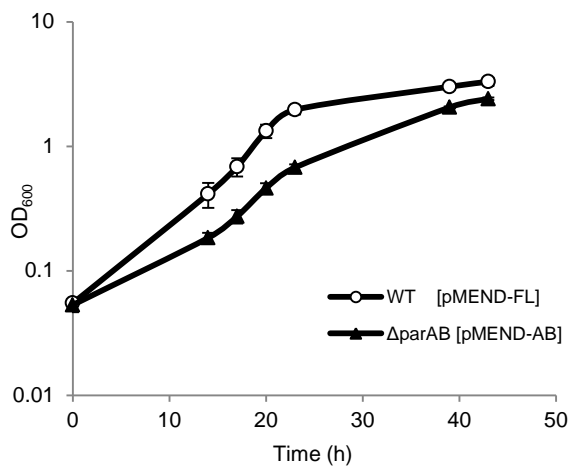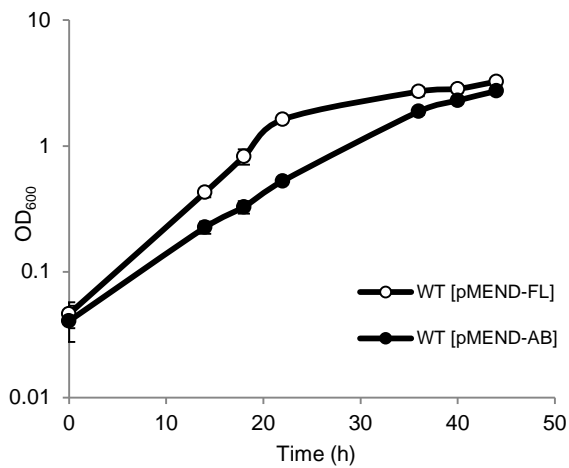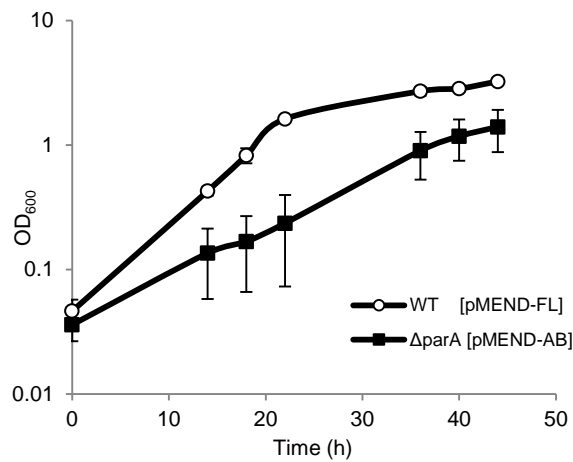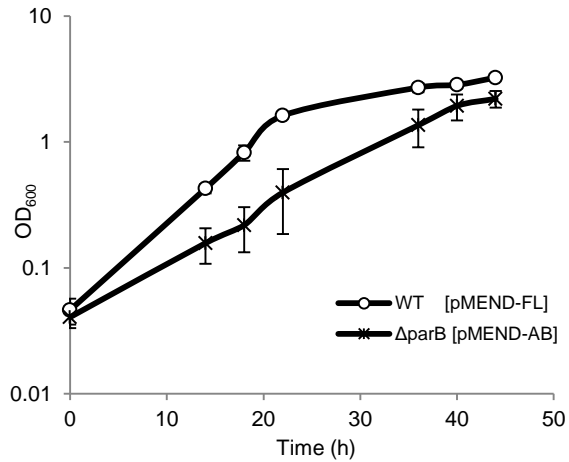

Supplement: S2 Fig — Strains were grown in Hartmans-de Bont medium and induced for the production of ParA-mCherry and ParB-EGFP. Growth curves were made with data collected from 3 biological replicates. Error bars indicate standard error of the mean. (PDF) [file pone.0199316.s002.pdf]

A. ParA and ParA-mCherry

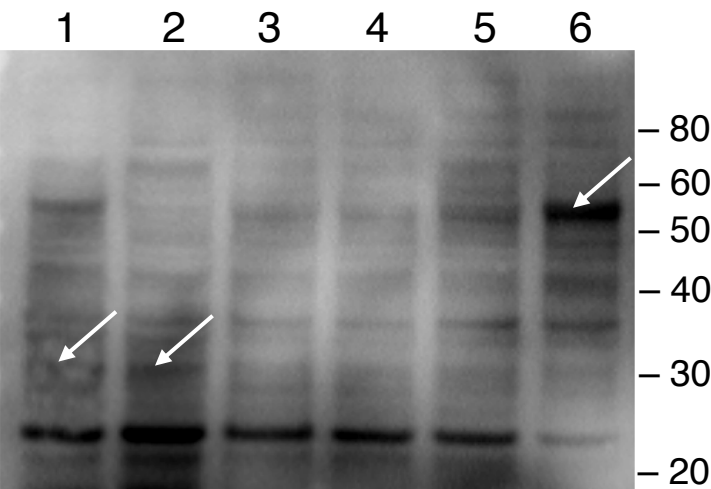

B. ParB and ParB-eGFP

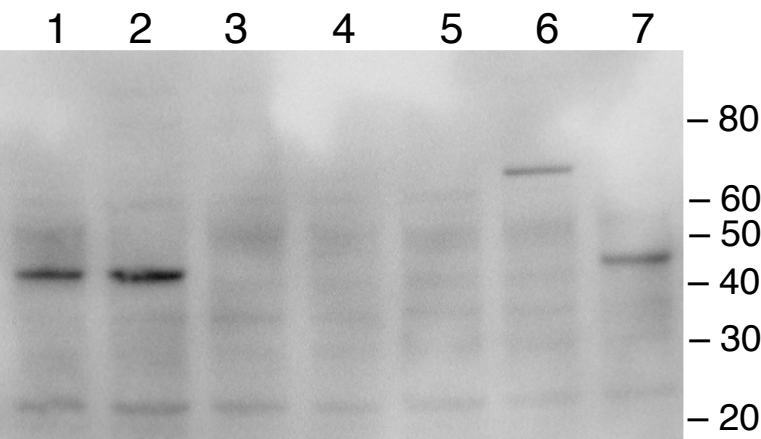

Supplement: S3 Fig — Cells were grown in the presence or absence of inducer. Panel A (1) wild-type, no inducer; (2) wild-type, plus inducer; (3) ΔparAB mutant, no inducer; (4) ΔparAB mutant, plus inducer; (5) ΔparAB [pMEND-AB), no inducer; (6) ΔparAB [pMEND-AB), plus inducer. ParA and ParA-mCherry bands are labelled with white arrows in wild-type and complemented strains. Panel B (1) wild-type, no inducer; (2) wild-type, plus inducer; (3) ΔparAB mutant, no inducer; (4) ΔparAB mutant, plus inducer; (5) ΔparAB [pMEND-AB], no inducer; (6) ΔparAB [pMEND-AB], plus inducer, (7) acetamide-induced ParB. (PDF) [file pone.0199316.s003.pdf]

Distance from mid-cell ( $\mu\text{m}$ )

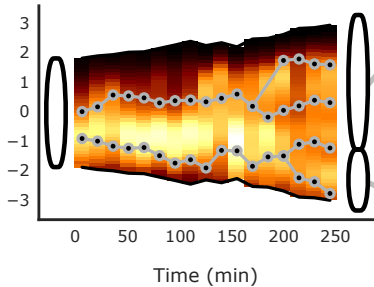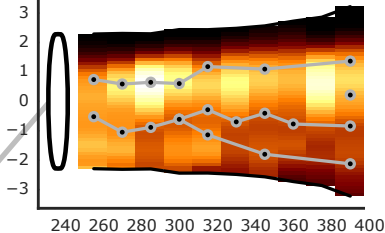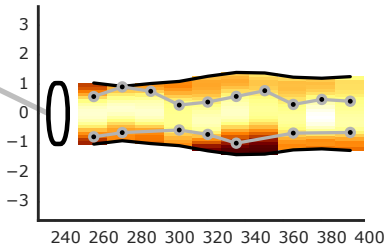

Supplement: S4 Fig — Dynamics are depicted as in Fig 3a. This figure represents a lineage of cells starting with a single cell which harbours two ParB-EGFP foci which each split into two foci before the excision of the cell into two daughter cells. In the upper daughter cell, one of the foci subsequently splits into two. (PDF) [file pone.0199316.s004.pdf]

**a**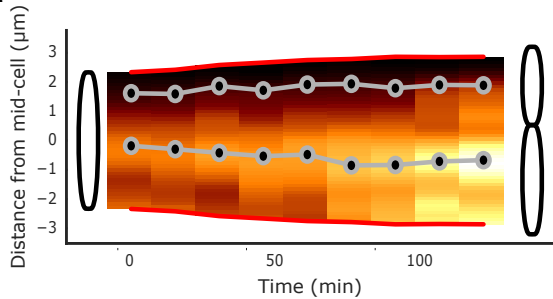**b**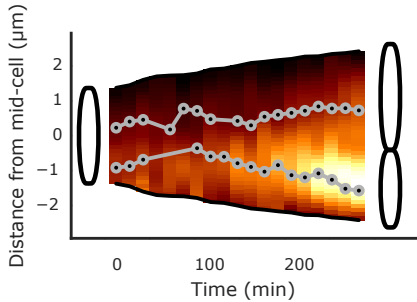

Supplement: S5 Fig — Dynamics are depicted as in Fig 3a. The new pole in the cell in panel (a) is unknown and this is indicated by both poles coloured in red. The new pole of the cell in panel (b) is situated at the bottom. This figure represents two independent cells in which ParB-EGFP foci have already split at the start of the visualisation period. Both cells divide into two daughters at the end of the period shown. (PDF) [file pone.0199316.s005.pdf]

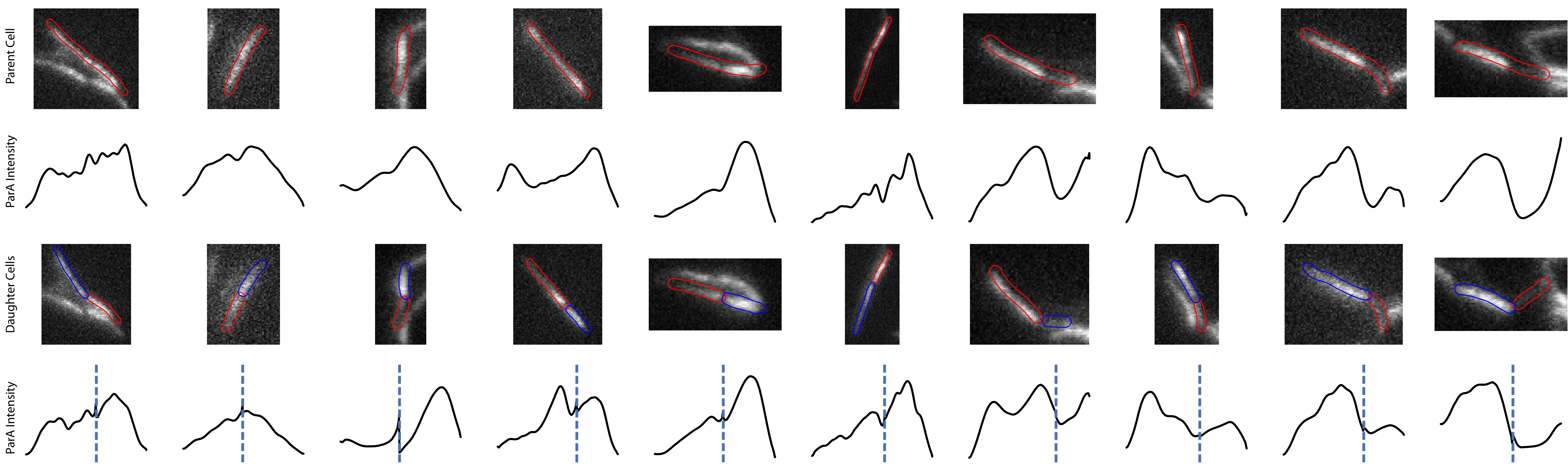

Supplement: S6 Fig — 10 cell divisions chosen at random are shown. The top row depicts the mother cell just before division, outlined in red. The second row shows the intensity profile along the cell axis for each mother cell. The third row shows the daughter cells post-division, outlined in blue and red. The bottom row shows the intensity profile for each of the daughter cells, with the division site shown as a blue dashed line. (PDF) [file pone.0199316.s006.pdf]
