# Supplementary material for: Analysis of ParAB dynamics in mycobacteria shows active movement of ParB and differential inheritance of ParA
Source: PLoS One. 2018 Jun 19;13(6):e0199316. doi: 10.1371/journal.pone.0199316 (PMC6007833; doi:10.1371/journal.pone.0199316)
Supplement: S1 Table — The values are defined and were measured as described in Methods. Mean values are represented ± the standard error of the mean. n = number of cells analysed to calculate each value. All strains were induced for the production of ParB-EGFP and ParA-mCherry. (PDF) [file pone.0199316.s007.pdf]

Strain	Doubling time (h)	Growth rate (h^{-1})	Division length (µm)
WT (no plasmid)	2.64 ± 0.15 ($n = 43$)	0.24 ± 0.01 ($n = 144$)	8.78 ± 0.19 ($n = 62$)
WT[pMEND-AB]	4.46 ± 0.35 ($n = 12$)	0.06 ± 0.01 ($n = 79$)	5.78 ± 0.31 ($n = 23$)
Δ*parAB*[pMEND-AB]	3.50 ± 0.24 ($n = 20$)	0.09 ± 0.01 ($n = 111$)	7.02 ± 0.30 ($n = 54$)
