# Supplementary material for: Analysis of ParAB dynamics in mycobacteria shows active movement of ParB and differential inheritance of ParA
Source: PLoS One. 2018 Jun 19;13(6):e0199316. doi: 10.1371/journal.pone.0199316 (PMC6007833; doi:10.1371/journal.pone.0199316)
Supplement: S2 Table — (PDF) [file pone.0199316.s008.pdf]

Strain / Plasmid	Description	Source
Escherichia coli DH5α	*F-, endA1, hsdR17 (rk-mk+), supE44, thi-1, recA1, gyrA, relA1, Δ(argF-lac)U169, deoRΦ80dlac, Δ(lacZ)M15*	[1]
M. smegmatis mc^{2}155	*ept-1*, mc^{2}6 mutant efficient for electroporation	[2]
M. smegmatis mc^{2}155::*parA*	*M. smegmatis* mc^{2}155 *MSMEG_6939::hygR*	This study
M. smegmatis mc^{2}155 Δ*parB*	*M. smegmatis* mc^{2}155 *ΔMSMEG_6938*	[3]
M. smegmatis mc^{2}155 Δ*parAB*	*M. smegmatis* mc^{2}155 *MSMEG_6939::hygR,ΔMSMEG_6938*	This study
pMEND-mCherry	Km^R, Hyg^R mycobacterial episomal expression vector pMEND containing *mcherry* under the control of a tetracycline inducible promoter.	[4]
pMEND-int	Integrative version of pMEND.	[5]
pMEND-mCherry-int	Integrative version of pMEND-mCherry.	This study
pMEND-FL	pMEND-mCherry-int containing the *riboswitch-egfp* 1278 bp fragment from plasmid pST5552.	This study
pMEND-A	pMEND-mCherry-int containing *parA* gene (*MSMEG_6939*) fused to the N-terminus of mCherry.	This study
pMEND-B	pMEND-mCherry-int containing the *riboswitch-parB-egfp* 2331 bp fragment from plasmid pST-B.	This study
pMEND-AB	pMEND-A containing the *riboswitch-parB-egfp* 2331 bp fragment from plasmid pST-B	This study
pST5552	Km^R mycobacterial episomal expression vector. Contains *egfp* under the control of a riboswitch-based theophylline inducible system.	[6]
pST-B	pST5552 containing *parB* (*MSMEG_6938*) fused to the N-terminus of EGFP.	This study
p2NIL	Km^R suicide vector backbone for allele replacement in mycobacteria.	[7]
pGOAL17	Ap^R *E. coli* vector containing PacI *lacZ* and *sacB* gene marker cassette.	[7]
p2NIL-parADel-HygR	p2NIL containing the *parA* gene sequence disrupted by the hygromycin cassette from plasmid pSE100.	This study
pAEV-ParA	Suicide vector for *parA* gene disruption in *M. smegmatis*. p2NIL-parADel-HygR with PacI gene marker cassette from pGOAL.	This study
pSE100	Hyg^R mycobacterial shuttle vector, source of hygromycin cassette.	[8]
pJV53	Km^R mycobacterial vector, containing the phage Che9c recombination genes 60-61 under the control of the acetamidase promoter. Episomal.	[9]

S2 Table References

1. Hanahan D. Studies on transformation of *Escherichia coli* with plasmids. J Mol Biol. 1983;166: 557–580.

2. Snapper SB, Melton RE, Mustafa S, Kieser T, Jr WRJ. Isolation and characterization of efficient plasmid transformation mutants of *Mycobacterium smegmatis*. Mol Microbiol. 1990;4: 1911–1919.

3. Jakimowicz D, Brzostek A, Rumijowska-Galewicz A, Żydek P, Dołzbłasz A, Smulczyk-Krawczyszyn A, et al. Characterization of the mycobacterial chromosome segregation protein ParB and identification of its target in *Mycobacterium smegmatis*. Microbiology. 2007;153: 4050–4060.

4. Joyce G, Williams KJ, Robb M, Noens E, Tizzano B, Shahrezaei V, et al. Cell division site placement and asymmetric growth in mycobacteria. PLoS One. 2012;7: e44582.

5. Williams KJ, Joyce G, Robertson BD. Improved mycobacterial tetracycline inducible vectors. Plasmid. 2010;64: 69–73.

6. Seeliger JC, Topp S, Sogi KM, Previti ML, Gallivan JP, Bertozzi CR. A riboswitch-based inducible gene expression system for mycobacteria. PLoS One. 2012;7: e29266.

7. Parish T, Stoker NG. Use of a flexible cassette method to generate a double unmarked *Mycobacterium tuberculosis tlyA plcABC* mutant by gene replacement. Microbiology. 2000;146: 1969–1975.

8. Guo X V, Monteleone M, Klotzsche M, Kamionka A, Hillen W, Braunstein M, et al. Silencing essential protein secretion in *Mycobacterium smegmatis* by using tetracycline repressors. J Bacteriol. 2007;189: 4614–4623.

9. van Kessel JC, Hatfull GF. Recombineering in *Mycobacterium tuberculosis*. Nat Meth. 2007;4: 147–152.
