# Supplementary material for: Analysis of ParAB dynamics in mycobacteria shows active movement of ParB and differential inheritance of ParA
Source: PLoS One. 2018 Jun 19;13(6):e0199316. doi: 10.1371/journal.pone.0199316 (PMC6007833; doi:10.1371/journal.pone.0199316)
Supplement: S3 Table — Restriction sites are underlined. (PDF) [file pone.0199316.s009.pdf]

Primer	Sequence (5'-3')	Use
parA roh F	tataaagctttcgagcgcaag	Cloning *parA* region of homology into p2NIL.
parA roh R	atcaagcttatcgagtgcacga	Cloning *parA* region of homology into p2NIL.
Hyg F	ctccatggtctgacagttaccaatgc	Cloning the hygromycin cassette from pSE100 into p2NIL-parADel.
Hyg R	atccatggagatgatcgaggatgcca	Cloning the hygromycin cassette from pSE100 into p2NIL-parADel.
MSMEG_6939 F	acgtggatccaggaggtgatgagcatgggttcgg	Cloning *MSMEG_6939* (*parA*) into pMEND-mCherry as a *parA-mcherry* fusion.
MSMEG_6939 R	taatcatatgctgctggcgcg	Cloning *MSMEG_6939* (*parA*) into pMEND-mCherry as a *parA-mcherry* fusion.
parB fus F (2)	ctaggaattcaatcagccggcacgcaag	Cloning *MSMEG_6938* (*parB*) into pST5552 as a *parB-egfp* fusion.
parB fus R	ctaggaattcctcgttctgggcgctcatc	Cloning *MSMEG_6938* (*parB*) into pST5552 as a *parB-egfp* fusion.
ParB-EGFP F	ccggttaattaactagaggtgaccacaacgc	Cloning the *riboswitch-parB-egfp* region from pST-B into pMEND-A.
ParB-EGFP R	ctagttaattaagcagacagttttattgttcatgatg	Cloning the *riboswitch-parB-egfp* region from pST-B into pMEND-A.
